# A comprehensive review on Compton camera image reconstruction: from principles to AI innovations

**DOI:** 10.1007/s13534-024-00418-8

**Published:** 2024-09-20

**Authors:** Soo Mee Kim, Jae Sung Lee

**Affiliations:** 1 Maritime ICT & Mobility Research Department, Korea Institute of Ocean Science and Technology, Busan, Korea; 2 Department of Nuclear Medicine, Seoul National University College of Medicine, 103 Daehak-ro, Jongno-gu, Seoul, 03080 Korea; 3 Brightonix Imaging Inc., Seoul, Korea

**Keywords:** Compton camera, Compton scatter, Image reconstruction, Gamma-ray, Single-photon imaging

## Abstract

Compton cameras have emerged as promising tools in biomedical imaging, offering sensitive gamma-ray imaging capabilities for diverse applications. This review paper comprehensively overviews the latest advancements in Compton camera image reconstruction technologies. Beginning with a discussion of the fundamental principles of Compton scattering and its relevance to gamma-ray imaging, the paper explores the key components and design considerations of Compton camera systems. We then review various image reconstruction algorithms employed in Compton camera systems, including analytical, iterative, and statistical approaches. Recent developments in machine learning-based reconstruction methods are also discussed, highlighting their potential to enhance image quality and reduce reconstruction time in biomedical applications. In particular, we focus on the challenges posed by conical back-projection in Compton camera image reconstruction, and how innovative signal processing techniques have addressed these challenges to improve image accuracy and spatial resolution. Furthermore, experimental validations of Compton camera imaging in preclinical and clinical settings, including multi-tracer and whole-gamma imaging studies are introduced. In summary, this review provides potentially useful information about the current state-of-the-art Compton camera image reconstruction technologies, offering a helpful guide for investigators new to this field.

## What is a Compton camera?

### Imaging principle

Non-invasive tomographic imaging techniques are widely used to observe meaningful functional and molecular changes inside living body by assessing the distribution of injected radiotracers. Single-photon emission computed tomography (SPECT) and positron emission tomography (PET) are conventional molecular imaging modalities utilizing radiotracers for various applications, such as neuroimaging, cancer diagnosis, drug discovery, and gene expression assessment [1–8]. The recent remarkable progress in high-performance imaging with these modalities can be attributed to the integration of functional and anatomical information, advances in computing power, and statistical image reconstruction algorithms [9–20].

**Fig. 1:**
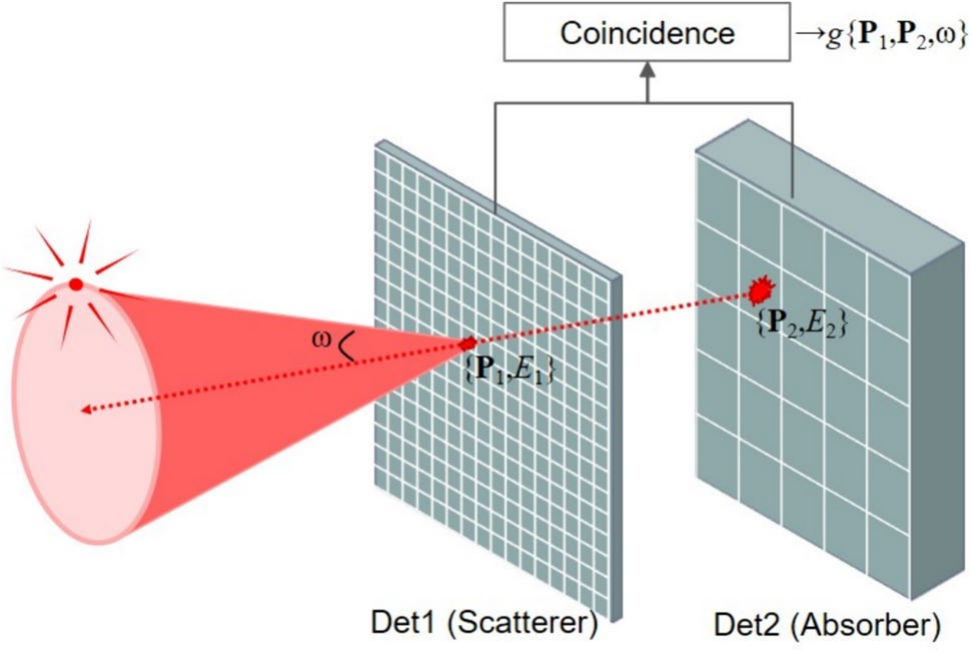
Coincidence detection of a typical Compton event with conical ambiguity (ω is the scattering angle and {**P**_1_, *E*_1_} and {**P**_2_, *E*_2_} are the positions and energies measured at the scatterer and absorber detectors, respectively)

An alternative molecular imaging modality, the Compton camera, typically consists of two position-sensitive radiation detectors with precise energy resolution (Fig. 1). The Compton camera provides a three-dimensional (3D) images of radiation source distribution by analyzing the measured signal of gamma rays that undergo successive interactions with two detectors—the scatterer and the absorber.

Conventional single-photon imaging systems, such as gamma cameras and SPECT, employ mechanical collimators that restrict the traveling pathway of gamma rays, resulting in a decrease in sensitivity. In contrast, the Compton camera utilizes the electronic collimation principle based on the relationship between the energy transfer and scattering angle of gamma rays. This electronic collimation endows the Compton camera with a great potential for higher sensitivity compared to other single-photon imaging modalities utilizing mechanical collimation. In a Compton camera, the incident gamma rays undergo Compton scattering at the first detector (scatterer) before being absorbed by the second detector (absorber), constituting valid events used for image reconstruction. A conical surface, specified by two interaction positions and a scattering angle derived from the interaction energy at the scatterer, represents the potential paths of these incident gamma rays, as illustrated in Fig. 1 [21–24]. The axis of the cone corresponds to the line connecting two interaction positions, **P**_1_(*x*,*y*,*z*) and **P**_2_(*x*,*y*,*z*), at the scatterer and absorber. The apex of the cone is the first interaction position (**P**_1_) in the scatterer, while the half-angle is equal to the scattering angle (*ω*) estimated from the measured interaction energies (*E*_1_ and *E*_2_).

### Advantages for biomedical applications

The Compton camera offers several potential advantages, including high-energy resolution and sensitivity. Because SPECT relies on mechanical collimation, it suffers from a tradeoff between detection efficiency and spatial resolution. In contrast, the Compton camera, based on electronic collimation, can overcome this limitation associated with mechanical collimation used in SPECT. Compton cameras are known to provide one or two orders of magnitude higher sensitivity than conventional SPECT cameras [24–26]. However, we need to consider that the uncertainty of the measured energy and the detection efficiency will decrease for low-energy gamma rays since the energy resolution and the sensitivity depend on the energy of the gamma rays to be detected. If the increased noise level due to conical back-projection of events for Compton camera image reconstruction is appropriately mitigated, the increased sensitivity can lead to the reduction of patient dose or scan time without loss of image quality.

The Compton camera is superior to PET in terms of simultaneous assessment of multiple radioisotopes. Although PET also uses electronic collimation, it generally cannot assess different radioisotopes simultaneously. This limitation arises because the energy of the annihilation gamma photons emitted from the positron emitters used in PET imaging is always the same (511 keV). Although multiplexed PET allows the simultaneous scan of two radiotracers [27], the application of this technique is limited to the positron emitters with prompt gamma emission. In contrast, the Compton camera can measure gamma rays with different energies simultaneously (also called multi-tracer imaging) because the radioisotopes used in the Compton camera are not limited to positron emitters. Furthermore, the excellent energy resolution of semiconductor detectors typically used in Compton cameras allows for high accuracy in discriminating different radioisotope distributions. Han et al. reported that a Si-based Compton camera provides a 20-fold improvement in detection efficiency for ^131^I (364 keV) compared to a gamma camera [26]. However, Leblanc et al. showed that the efficiency of the Compton camera diminishes for ^99m^Tc imaging [25].

Sections 2 and 3 introduce recent advancements in hardware and image reconstruction algorithm developments for the Compton camera aimed at achieving high resolution and sensitivity. In addition, Sect. 4 provides a summary of the biomedical applications envisioned for the future Compton camera. Because detailed reviews on Compton camera systems and their biomedical applications are available elsewhere [28, 29], we have primarily focused on image reconstruction algorithms and technologies. However, it is important to acknowledge that this review has inherent limitations. Due to the constraints in space and knowledge, this review may not encompass all relevant studies in this field. Therefore, while striving for comprehensiveness, this review remains a snapshot of the current situation, recognizing the dynamic nature of research and innovation in Compton camera image reconstruction and biomedical applications.

## Brief history of compton camera development for biomedical applications

Pinkau and White introduced the principles of the Compton camera for imaging solar neutrons [30, 31]. One of the most successful applications of the Compton camera is its application to the Compton telescope for gamma-ray astronomy [32–34]. However, since the focus of this paper is on the development of the Compton camera for biomedical applications, we will not go into the details of the Compton telescope.

In 1974, Todd and Everett discussed the feasibility of the Compton camera for medical applications compared to Anger cameras equipped with mechanical collimators [21]. Singh et al. reported analytical and experimental results with the first prototype of the Compton camera for medical applications [35–37]. Their Compton camera consisted of a high-purity germanium (HPGe) detector and a conventional Anger camera as scatter and absorber detectors, respectively. In subsequent investigations, semiconductor detectors and NaI scintillators were typically chosen for the scatterer and absorber detectors, respectively. Martin and Gormley proposed the combination of an HPGe detector array and a NaI scintillator ring and demonstrated the feasibility of the Compton camera for various radiation detection applications [24, 38]. Alternative combinations, such as Si pad detectors and NaI scintillators in C-SPRINT, were proposed and compared to SPECT systems in terms of sensitivity and resolution [39, 40].

The advent of strip solid-state detectors led to a compact design and the enhanced energy resolution of Compton cameras [41–43]. Compton cameras with double-sided strip silicon detector (DSSD) and segmented Ge detector or scintillation detector as scatterer and absorber were developmental, and their system parameters were optimized through simulations and experiments [44–47]. The Ge detector provided superior energy resolution but required significant system cooling. Alternatively, cadmium telluride (CdTe) and cadmium zinc telluride (CdZnTe or CZT), which have been utilized for clinical single-photon imaging systems [48, 49], have also been adopted in Compton cameras because they operate at room temperature [50–53]. In particular, 3D position-sensitive Compton cameras have the advantage of compact size, large field of view, and high spatial resolution [54, 55].

The evolution of scintillation crystals with high stopping power and excellent energy resolution, including cerium-doped gadolinium aluminum gallium garnet (Ce:GAGG), and cerium-doped lanthanum bromide (Ce:LaBr3), has led to the emergence of novel scintillation detector-based Compton cameras. These advances are also attributed to introducing a novel photosensor, silicon photomultiplier (SiPM) [56–61]. This semiconductor-based photosensor offers many advantages over conventional photomultipliers, including high quantum efficiency and insensitivity to magnetic fields, consequently driving significant advancements in PET and associated hybrid imaging systems [62, 63]. The compactness of SiPMs allows fine spatial resolution and high light collection efficiency, both of which are critical in scintillation detectors for Compton cameras [64].

Tracking the recoil electrons generated during the Compton scattering process significantly reduces background noise in reconstructed images by providing information about the incident gamma ray's direction [65, 66]. This direction information, obtained by measuring the recoil electron’s trajectory, mitigates ambiguity in determining the origin of detected gamma rays. Although gaseous detectors exhibit superior recoil electron tracking performance due to reduced multiple scattering, their drawback lies in their low gamma-ray stopping power. Semiconductor and scintillation detectors present promising alternatives to gaseous detectors for electron tracking, offering higher detection efficiency [67, 68].

Various system designs were proposed to increase spatial resolution and sensitivity, including multi-layered scatterer designs or spherical designs [69–73]. While most systems consisted of single or multi-scatterer and absorber detectors arranged in parallel, C-SPRINT and UCL Compton cameras were configured in a ring-type structure.

Numerous studies have demonstrated the feasibility of the Compton camera for medical imaging. However, challenges in increasing spatial resolution and sensitivity persist for practical applications. Lee et al. compared three different semiconductor detectors (Si, Ge, and CdTe) using Monte Carlo simulations regarding energy resolutions and efficiencies of Compton scattering and absorption interactions [74]. Si offers high energy resolution and efficiency of single and multiple Compton scattering interactions, making it an appropriate scatterer for determining more accurate scattering angles and increasing the number of Compton-scattered events. CdTe provides superior performance with respect to the detection efficiency of scattered gamma rays. Kabuki et al. reported that combining Compton-MR imaging achieved an energy resolution of 14% and a spatial resolution (FWHM) of 11 mm at 10 cm for 511 keV [75].

## Image reconstruction for compton camera

Compton data, *g*(**P**_1_,**P**_2_,*ω*), measured at two successive interaction positions (**P**_1_ and **P**_2_) on the scatterer and the absorber with a scattering angle (*ω*) can be expressed through a conical surface integral of a given source distribution function. The cone is formed with an axis connecting two interaction positions (**P**_1_ and **P**_2_) and a half-angle (*ω*), as illustrated in Fig. 1. Various methods have been proposed to reconstruct the 3D source distribution from the Compton-scattered projection data [76].

### Simple back-projection method

**Fig. 2:**
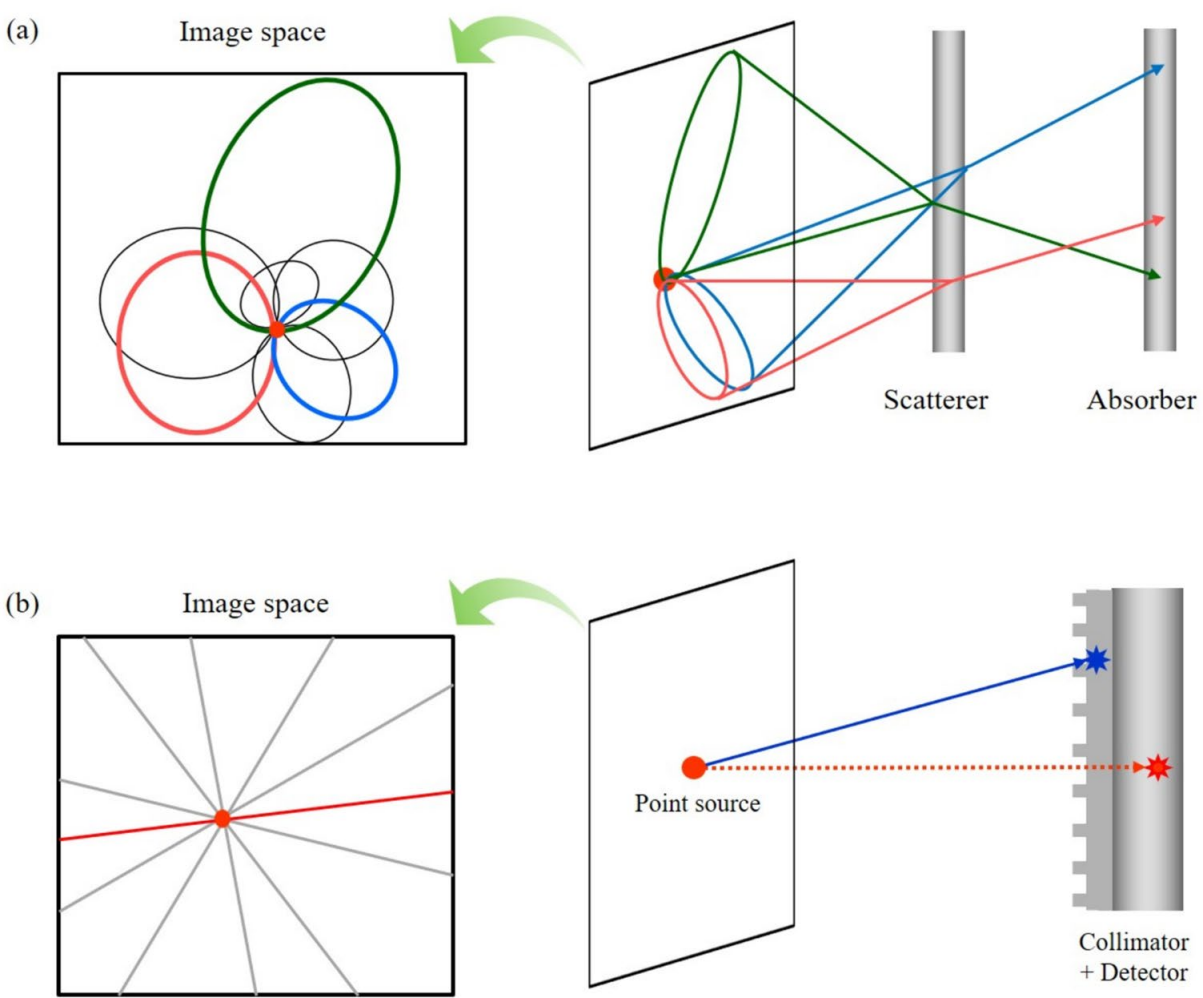
Simple back-projection: super-imposed **a** cones and **b** lines back-projected from the measured data into image space

The simplest method to reconstruct an image from Compton-scattered projections is by back-projecting them into a 3D image space along the conical surfaces, as illustrated in Fig. 2a [77–79]. Unlike other imaging systems, such as SPECT and PET, which rely on the line integrals (Fig. 2b), the back-projection images of a Compton camera are obtained by superimposing ellipses on cross-sectional slices assuming no back-scattered events. The more Compton data measured, the more ellipses are overlapped, resulting in higher signal intensity.

**Fig. 3:**
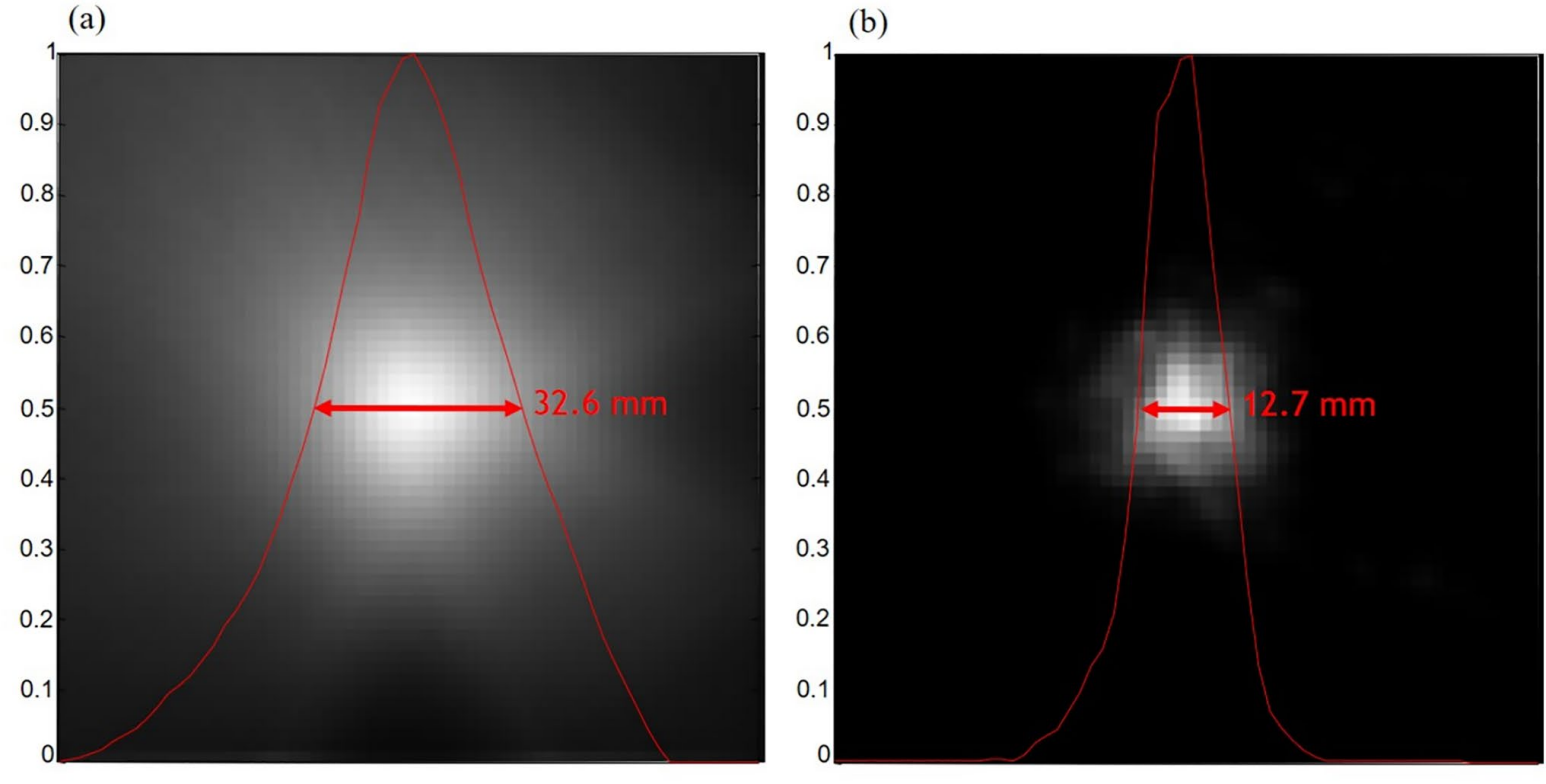
Comparison of the reconstructed images of a simulated point source using **a** simple back-projection and **b** OSEM (from [81]; with permission)

Simple back-projection has been widely used in astronomical applications because it is a straightforward method. However, biomedical applications require more sophisticated reconstruction methods because simple back-projection significantly reduces edge-sharpness and source-to-background contrast. Relatively higher background levels around pathologic lesions in biomedical data further aggravate these shortcomings of the simple back-projection method. Figure 3 compares the reconstructed images using the simple back-projection and ordered-subset expectation maximization (OSEM), a statistical image reconstruction method, for simulation data of a source point (diameter = 1 mm). Simple back-projection degrades the background pixels around the actual position of a point source. Rohe et al. adopted additional deconvolution with spatially variant point kernels to deblur the image obtained by simple back-projection [80]. The spatially variant point kernels were derived through the simulations of several point sources and the spherical Compton camera geometry.

### Rebinning compton-scattered data

**Fig. 4:**
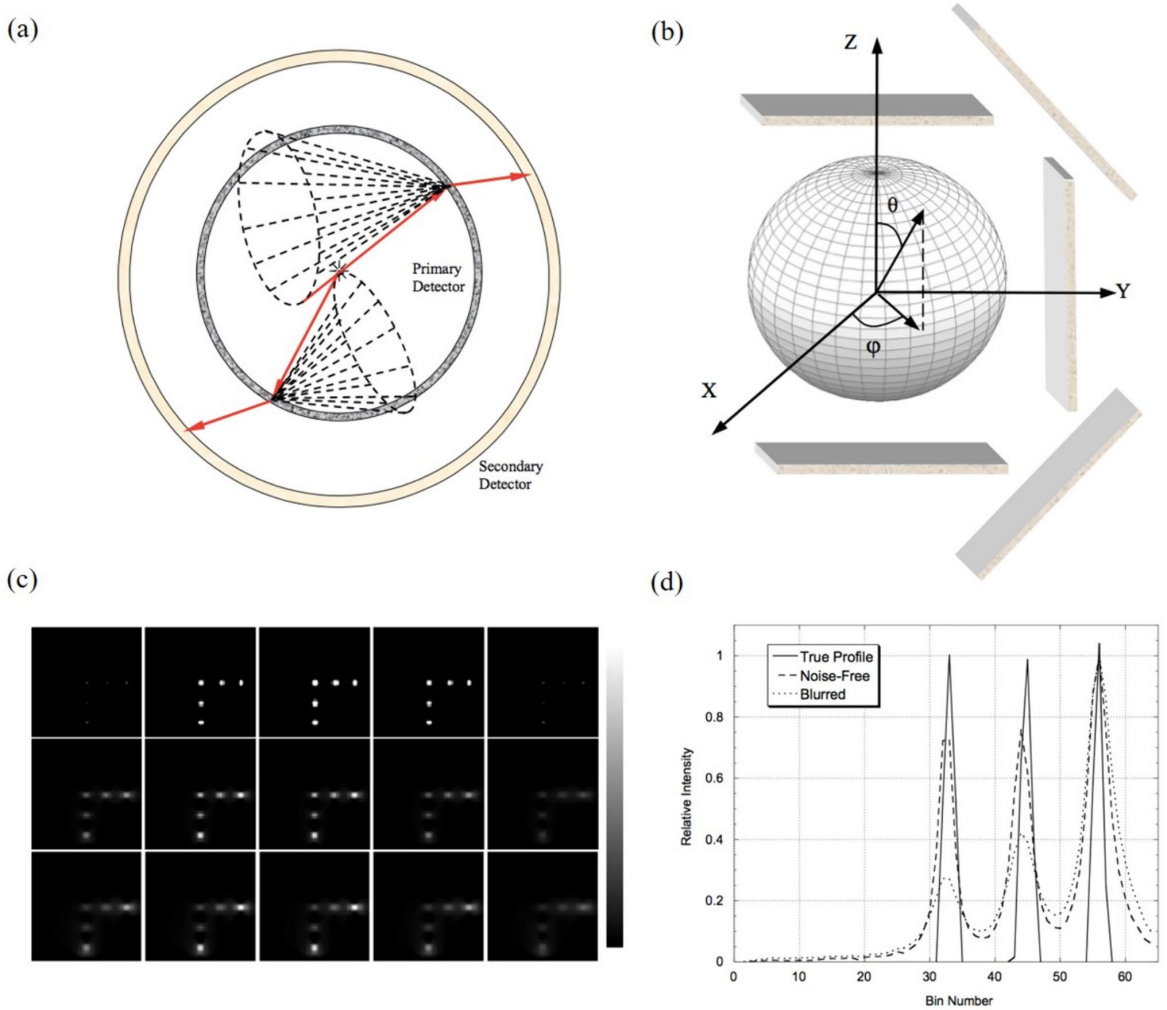
Rebinning the Compton scattered projection data : (**a**) equally spaced lines on the conical surfaces, (**b**) a spherical coordinate system, (**c**) FBP images from the rebinned simulation data of 5 point sources, and (**d**) the profiles between the ground truth and reconstructed images (from [82]; with permission)

The rebinning method of Compton data into the parallel projection, similar to SPECT, has been proposed [82, 83]. This method divides the cone surface corresponding to measured Compton data into a number of equally spaced straight lines, as shown in Fig. 4a. The orientation of each sampled line is then defined, and these lines are sorted according to polar and azimuthal angles (*θ* and *φ*) in a spherical coordinate system in Fig. 4b. If a sampled line on the cone surface, indicated by the red line in Fig. 4a, has a direction vector, $\overrightarrow{D}=\left({D}_{x},{D}_{y},{D}_{z}\right)$, its corresponding orientation can be calculated as:

1 $$ \theta = \cos^{ - 1} \left( {\frac{{D_{z} }}{{\sqrt {D_{x}^{2} + D_{y}^{2} + D_{z}^{2} } }}} \right)\;{\text{and}}\;\varphi = \cos^{ - 1} \left( {\frac{{D_{x} }}{{\sqrt {D_{x}^{2} + D_{y}^{2} } }}} \right) $$

The sorted sampling lines projected onto the virtual detector planes perpendicular to the lines at *θ* and *φ* form typical parallel projections, which are subject to traditional image reconstruction algorithms such as filtered back-projection (FBP) and algebraic reconstruction technique (ART) used for conventional tomography systems. Figure 4c shows the FBP images reconstructed from the rebinned simulation data of 5 evenly spaced point sources, and Fig. 4d compares the profiles between the ground truth and reconstructed images under various simulation conditions. Notably, nonuniform intensity and blurriness are evident both within and across the slices.

While the rebinning method alleviates the complexity of full 3D computation of conical surface integration and reconstruction problems, several practical challenges should be addressed:

(i) Optimization of weighting in the sampling step of conical surface-to-line projections.
(ii) Determining the optimal configuration of virtual detector planes to balance the tradeoff between spatial resolution and computation load.
(iii) Improving interpolation methods and normalization in the summation step for the final reconstructed image.
(iv) Handling truncated angular coverage over 3D image space in the non-spherical geometry of realistic Compton cameras, such as two detector planes or cylindrical scanners.

### Analytical image reconstruction methods

Efforts spanning numerous studies [84–94] have been undertaken to explore direct analytical image reconstruction methods for Compton cameras. Specifically, the projection data at the second detection location **D** (**P**_2_ in Fig. 1) and scattering angle ($\upomega$) is expressed through the integrals of the source distribution, $f\left(\mathbf{S}\right)$, over all possible conical surface:

2 $$ g\left( {{\mathbf{D}},{\omega }} \right) = K\left( {\omega } \right)\iint {\delta \left( {cone} \right)f\left( {\mathbf{S}} \right)d{\mathbf{S}}\frac{1}{{{\mathbf{SM}}^{2} }} \times dx_{M} dy_{M} \frac{1}{{l^{2} + x_{M}^{2} + y_{M}^{2} }}} $$

where *K* is determined by the Klein-Nishina formula, **M** represents all the possible first detection position (**P**_1_ in Fig. 1), and *l* denotes the distance between the scatter and absorber. However, direct inversion for reconstructing the source distribution based on this general formulation appears challenging.

One class of analytical reconstruction methods for the Compton camera employs spherical harmonics to devise the inversion technique of Eq. (2), converting the Compton cone-surface projections into cone-beam projections placed onto the cone surface. This involves defining the inverse point spread function (PSF) in spherical harmonics for a given detector element, enabling the construction of cone-beam projections with cones of all scattering angles sharing the same cone axis through deconvolution in spherical coordinates. The line projections, $g\left( \Omega \right)$, are then computed as the convolution of a simply back-projected image, $g^{\prime}\left( {\Omega^{\prime}} \right)$, through cones defined by all possible scattering directions ($\Omega_{2} , \omega_{2}$) toward the second detector and inverse PSF, $h^{ - 1} \left( {\cos \omega } \right)$:

3 $$ g\left( \Omega \right) = \int g^{\prime}\left( {\Omega^{\prime}} \right)h^{ - 1} \left( {\cos \omega } \right)d\Omega^{\prime} $$

The inverse PSF can be expressed through an expansion of the Legendre polynomials in terms of spherical harmonics:

4 $$ h^{ - 1} \left( {\cos \omega } \right) = \mathop \sum \limits_{n = 0}^{\infty } \left( {\frac{2n + 1}{{4\pi }}} \right)^{2} \frac{{P_{n} \left( {\cos \omega } \right)}}{{H_{n} }} $$

Here, the coefficients of Legendre polynomials, $H_{n}$ are computed as following equation where $P_{n} \left( {\cos \omega } \right)$ represents the basis functions of Legendre polynomials.

5 $$ H_{n} = \frac{2n + 1}{2}\int {h\left( {\cos \omega } \right)P_{n} \left( {\cos \omega } \right)d\left( {\cos \omega } \right)} $$

Cree and Bones [84] and Basko et al. [85] have developed direct analytical reconstruction methods under simple assumptions, such as a very limited acceptance angle (only 90°) on the second detector or neglecting the frequency distribution of scattering angles (Klein-Nishina distribution).

Parra [86] proposed a dedicated FBP algorithm for a Compton camera which accommodates complete data from all scattering angles and considers measurement error. Parra's method allocates every detector element in the first detector into a unit spherical space, where the specific event response functions of measured scattering angles for a given detector element are summed. Through the summation of event response functions for all detector elements, the cone beam projections are generated as in Eq. (3) and then reconstructed using traditional cone beam reconstruction algorithms. Extensions of Parra’s method have been developed for limited scattering angles [87], taking into account resolution loss due to the broadening effect on scattering angles [88].

**Fig. 5:**
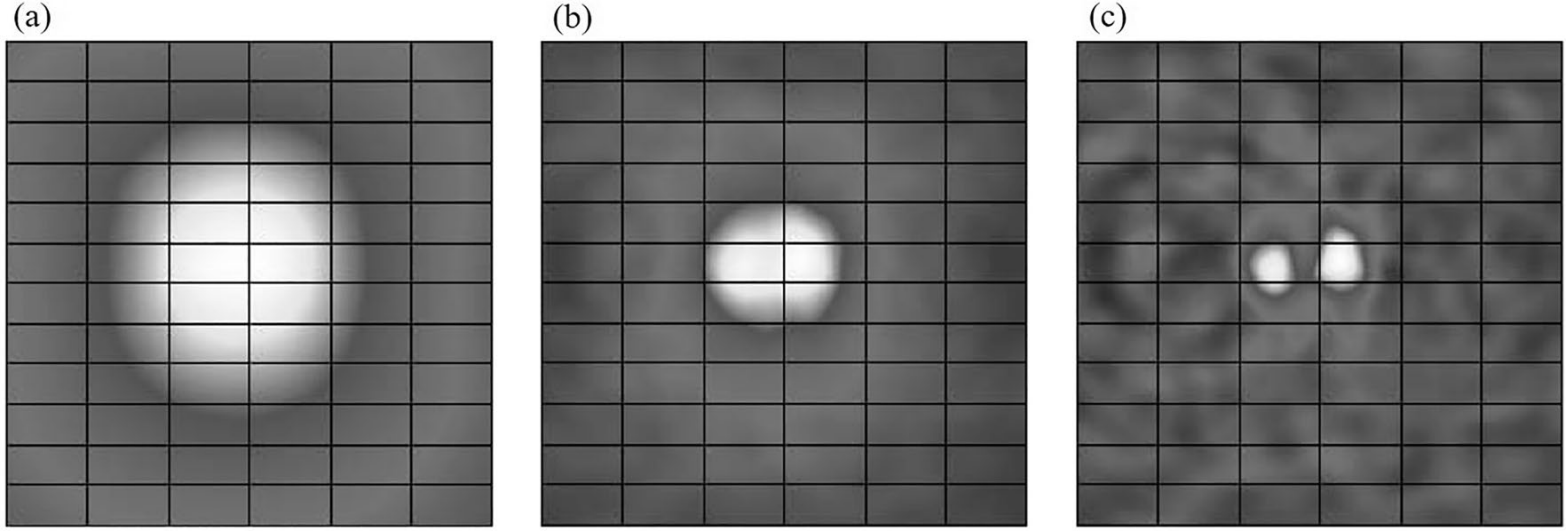
Reconstructed images of the actual measurements from two.^137^Cs sources: (**a**) simple back-projection, (**b**) FBP with a theoretical PSF, (**c**) FBP with the simulated PSF. (from [89]; with permission)

Xu et al. reported discrepancies between the simulated scattering angle distribution of 662 keV gamma rays on their CdZnTe detector and the theoretical prediction based on the Klein-Nishina formula. They applied an FBP with a more accurate PSF simulated for 4π Compton Imaging [89]. Figure 5 shows the reconstructed images of measured Compton data (40,000 events) from two ^137^Cs point sources (10 μCi) separated by 15 degrees. In this figure, FBP with the simulated PSF produced improved results, distinguishing between the two points in the reconstructed image, unlike simple back-projection and FBP with the theoretical PSF.

Another analytical reconstruction is related to different inversion techniques of conical Radon transform in Eq. (2). Truong et al. proposed an analytic inversion method for the special $\mathcal{C}$_2_-Radon transform, which proves invertible under the condition that the axis of a cone is perpendicular to the detector surface [90]. Maxim et al. subsequently presented a simplification of Eq. (2) as follows:

6 $$g\left({u}_{1},{u}_{2};\,\,\alpha ,\delta ,\omega \right)=K\left(\omega \right)\int \delta (cone)f\left(\mathbf{S}\right)cos\theta d\mathbf{S}$$

$\alpha$ and $\delta$ represent the polar and azimuthal angles of the cone axis, respectively, in a spherical coordinate system. $\theta$ denotes the incidence angle into the first detector. Analytic inversion formula can be derived from Eq. (6) utilizing the complete set of available projections [92, 95]:

7 $$ \mathcal{H}\mathcal{F}\left\{ f \right\}\left( {\eta_{1} ,\eta_{2} ,\eta_{3} } \right) = \frac{1}{{K\left( {\omega } \right)b\left( {{\alpha },{\omega }} \right)}}\mathcal{F}_{2} \left\{ g \right\}\left( {\eta_{1} ,\eta_{2} ;\alpha ,\delta ,{\omega }} \right) $$

where $$b\left( {{\upalpha },{\upomega }} \right) = \frac{sin\omega }{{\sqrt {cos^{2} \alpha - sin^{2} \omega } }}$$.

Equation (7), which is the central-slice theorem for the inversion of the Compton transform, indicates that the 2D Fourier transform of the cone-surface projection, *g* is proportional to the Hankel-Fourier transform of the intensity function *f*. Lojacono et al. proposed a new formula of FBP derived from Eq. (7) tailored for the computer implementation [91, 93]:

8 $$ f\left( {x,y,z} \right) = 2\pi \mathop \int \limits_{0}^{\pi } \mathop \int \limits_{ - \infty }^{\infty } \left( {\mathop \smallint \limits_{0}^{\infty } \mathcal{F}_{\tau ,\delta }^{ - 1} \left\{ f \right\}\left( \rho \right)J_{0} \left( {2\pi z\tau \rho } \right)d\tau } \right) \times e^{{2i\pi \rho \left( { - xsin\delta + ycos\delta } \right)}} \left| \rho \right|^{3} d\rho d\delta $$

These analytical reconstruction methods utilizing spherical harmonic expansion or direct inversion formula of the conical Radon transform offer advantages in computation time. However, they do not ensure the suppression of statistical fluctuations in the measured data due to the inherent randomness of emission and detection processes. In addition, there are concerns regarding algorithmic stability, particularly in addressing ill-posedness [76].

### Statistical image reconstruction methods

While analytical reconstruction algorithms for the Compton camera are useful for achieving image reconstructions within a clinically acceptable time, statistical reconstruction algorithms, such as maximum likelihood (ML) and maximum a posteriori (MAP) approaches, enhance the accuracy of Compton camera reconstruction [76]. These statistical reconstruction algorithms, that have demonstrated their efficacy in conventional emission computed tomography [11], can be readily adapted to Compton camera reconstruction.

#### ML approach

The ML approach for the Compton camera is based on the Poisson likelihood probability of the detection process relating to conical integration, as expressed in Eq. (9), which results in an estimate of the source distribution by maximizing the likelihood:

9 $$ {\hat{\mathbf{f}}} = \arg \mathop {\max }\limits_{{\mathbf{f}}} {\text{Pr}}\left( {{\mathbf{G}} = {\mathbf{g}}|{\mathbf{f}}} \right) $$

A popular method to solve the ML estimation problem is the Expectation Maximization (EM) algorithm, denoted as MLEM [96, 97]. The MLEM algorithm reconstructs an image of the source distribution through an iterative process that involves a forward projection of the estimated source distribution and a backward projection of the ratio between the measured and estimated projection data as the following equation:

10 $$ \hat{f}_{i}^{k + 1} = \frac{{\hat{f}_{i}^{k} }}{{\mathop \sum \nolimits_{j} H_{ij} }}\mathop \sum \limits_{j} H_{ij} \frac{{g_{j} }}{{\mathop \sum \nolimits_{l} \hat{f}_{l}^{k} H_{lj} }} $$

A mathematical expression for Compton projection data at *j*^th^ event $\left( {{\mathbf{P}}_{1} ,{\mathbf{P}}_{2} ,\omega } \right)$ is given in Eq. (11):

11 $$ g_{j} = \mathop \sum \limits_{i} H_{ij} f_{i} $$

**Fig. 6:**
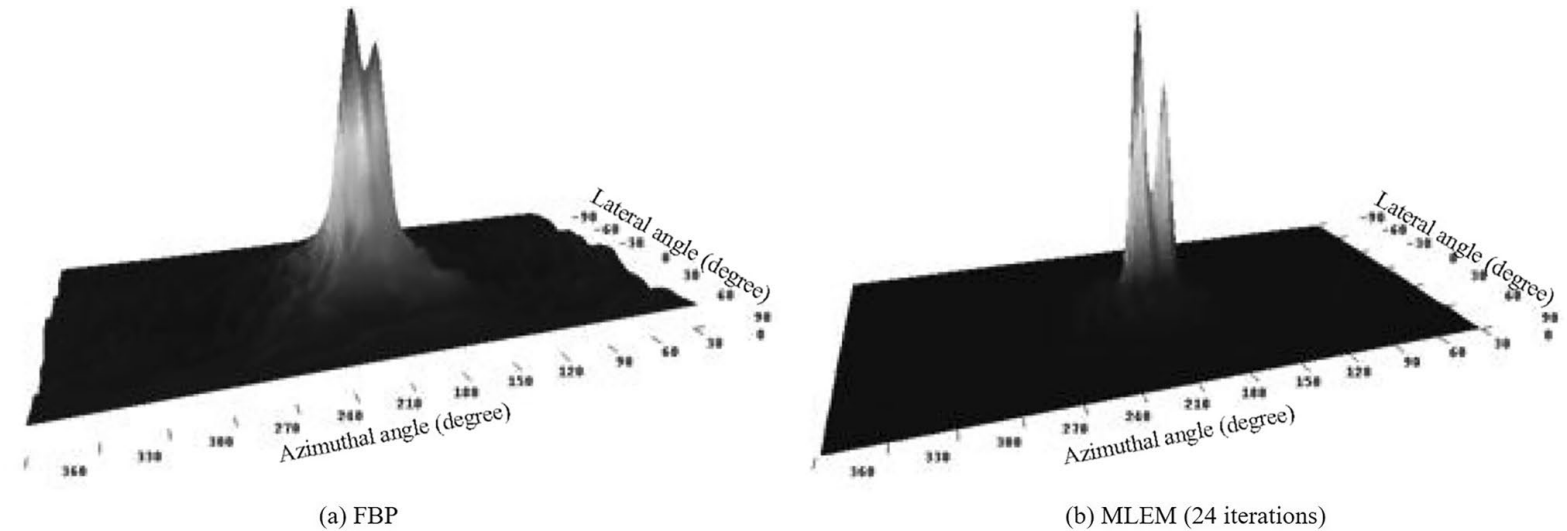
Reconstructed images of two.^137^Cs sources separated by 15 degrees using (**a**) FBP and (**b**) MLEM. (from [101]; with permission)

Here, the system matrix, ${H}_{i;j}$ represents the probability that a photon emitted from *i*^th^ voxel is scattered at position ${\mathbf{P}}_{1}$ on the scatterer with a scattering angle $\omega$ and detected at position ${\mathbf{P}}_{2}$ of the absorber. Kim et al. proposed two approaches to effectively compute the system matrix: the ellipse-stacking method (ESM) and the ray-tracing method (RTM) [98]. In ESM, the measured cone surfaces are sampled by the image planes parallel to the scatterer, and the belonging probabilities are computed based on the proximity of the intersected voxels and the ellipse in the image plane. In RTM, the belonging probability is approximated by the intersecting length of the voxels and the sampled rays passing through the apex on the conical surface. MLEM has been applied to both binned and list-mode data of the Compton cameras [99, 100]. Xu et al. reported that MLEM with 24 iterations outperformed Parr’s FBP in distinguishing two ^137^Cs point sources placed 15 degrees apart (Fig. 6) [101].

Due to the slow convergence rate of MLEM, which requires a significant number of iterations, accelerated reconstruction algorithms have been explored. One such method is the Ordered Subsets EM (OSEM) algorithm, initially proposed by Hudson and Larkin and widely utilized in commercial SPECT and PET systems [102]. OSEM applies MLEM to each subset of projection data. OSEM has been adapted for Compton-scattered data, and subset ordering schemes have been optimized to allow larger and more uniform angular and positional differences in allocating subsets based on scattering angles and detected positions [103–105]. Remarkably, for an event number of two million, OSEM with 16 subsets achieved a remarkable speed-up of 9.5 times compared to MLEM with 256 iterations.

The row-action ML algorithm (RAMLA) is an OS algorithm developed to address convergence instability by incorporating a relaxation parameter [106]. This method has been enhanced for the Compton camera by taking into account the system's geometric characteristics and selecting relaxation parameters inversely proportional to the system matrix [107]. Since counting statistics in real systems is dependent on scan time, most reconstruction algorithms based on ML estimation are susceptible to statistical fluctuations in measured data.

#### MAP approach

The MAP approach is a statistical method used to solve estimation problems by maximizing the a posteriori probability of the parameters given the observed data:

12 $$ {\hat{\mathbf{f}}} = \begin{array}{*{20}c} {\text{argmax}} \\ {\mathbf{f}} \\ \end{array} {\text{Pr}}\left( {{\mathbf{F}} = {\mathbf{f}}{|}{\mathbf{G}} = {\mathbf{g}}} \right) = \begin{array}{*{20}c} {\text{argmax}} \\ {\mathbf{f}} \\ \end{array} \left[ {{\text{Pr}}\left( {{\mathbf{G}} = {\mathbf{g}}{|}{\mathbf{F}} = {\mathbf{f}}} \right) + {\text{Pr}}\left( {{\mathbf{F}} = {\mathbf{f}}} \right)} \right] $$

In Eq. (12), the a posteriori probability is expressed by combining likelihood and prior probabilities via the Bayes’ theorem. The prior probability (sometimes referred to as penalty function), Pr(**F** = **f**) in Eq. (12), represents the local information regarding source distribution used to suppress the instability of the ML estimation approach due to statistical noise.

Block Sequential Regularized EM (BSREM) and relaxed OS separable parabolic surrogates (rOS-SPS) are reconstruction algorithms that incorporate ordered subsets strategies into MAP approaches for accelerating image reconstruction speed [108, 109]. These methods iteratively update the image reconstruction by dividing the projection data into subsets, processing them sequentially, and incorporating regularization techniques to improve image quality and reduce noise. In addition, Penalized Weighted Least Squares (PWLS) and BSREM algorithms have been successfully applied with convex-nonquadratic penalty functions, which serve as edge-preserving regularization terms, for reconstructing Compton-scattered data [110, 111]. These methods aim to enhance the quality of reconstructed images by incorporating penalties that preserve image features while reducing noise and artifacts.

#### Advanced implementation techniques

While applying ML and MAP approaches to Compton camera reconstruction may pose challenges due to the heavy computational load compared to analytic reconstruction algorithms, the advancing high-performance computing techniques make these approaches more practical. For instance, parallel computing techniques utilizing graphics processing units (GPUs) have been successfully applied to Monte Caro simulation and image reconstruction for the Compton camera [112–114], offering significant improvements in computational efficiency and speed.

**Fig. 7:**
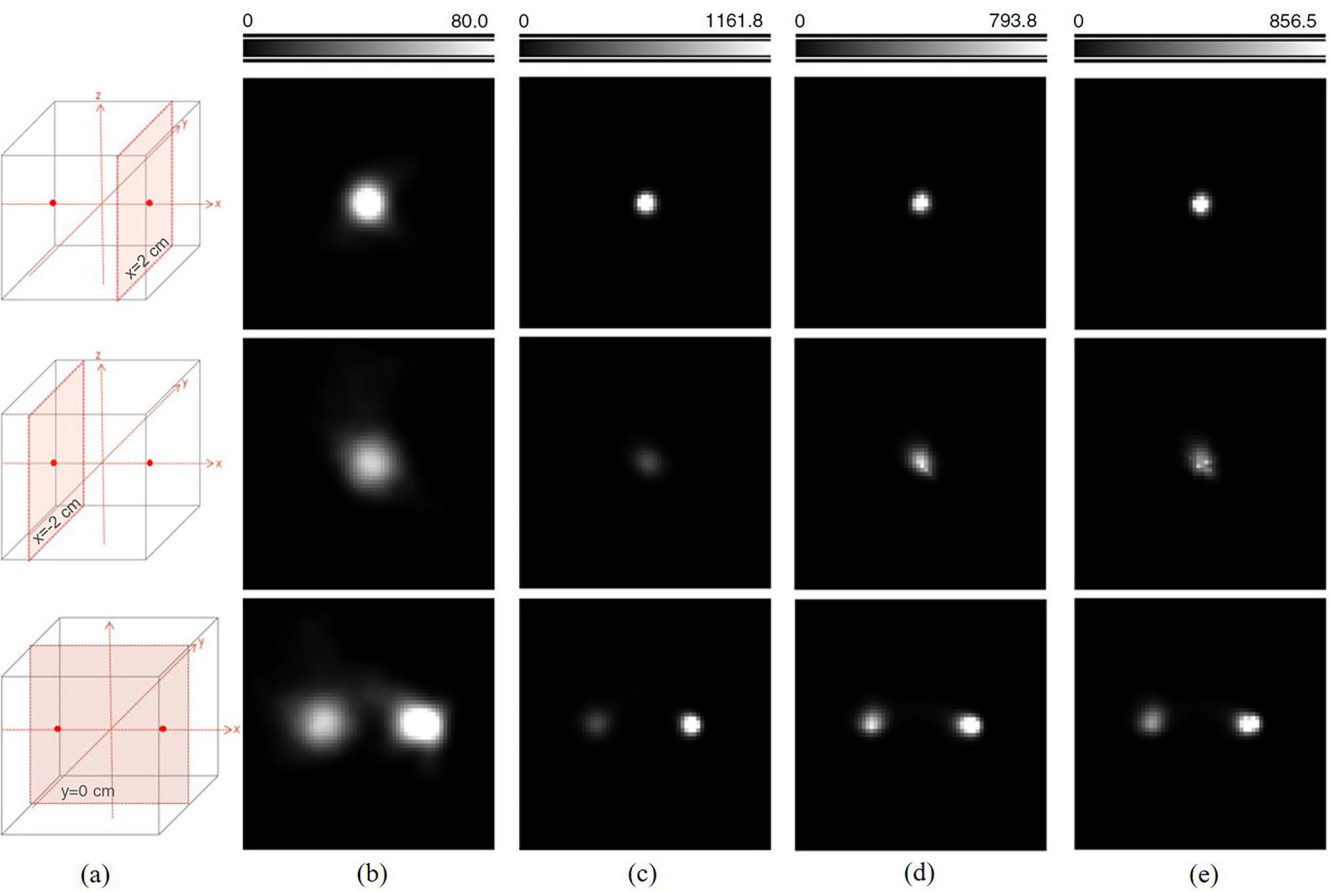
Effects of resolution recovery during statistical image reconstruction. (**a**) Simulated point source position, and reconstructed images using LMOSEM (**b**) without PSF model, (**c**) space-invariant symmetric Gaussian kernel, (**d**) space-variant asymmetric Gaussian kernel, and (**e**) space-variant symmetric general PSF. (from [81] with permission)

In Compton camera imaging, the spatial resolution is influenced by uncertainties in determining scattering angles and detected interaction position pairs which define the cones [115]. Various factors, including limited energy resolution, Doppler broadening, and detector element size, contribute to angular and positional uncertainties in measurements [116–119]. These uncertainties impact the surface thickness and axis location of the cone defined by the measured positions and scattering angles. To address this issue, Kim et al. incorporated the PSF of the Compton camera into the system matrix of statistical image reconstruction to achieve resolution recovery (RR) during image reconstruction [81]. Their proposed method, the list-mode Ordered Subsets Expectation Maximization (LMOSEM) incorporating the shift-variant RR model, led to a remarkable 2.6-fold improvement in spatial resolution compared to standard LMOSEM without RR modeling (Fig. 7). Moreover, the proposed method outperformed LMOSEM with a uniform RR model in terms of spatial resolution uniformity. These advancements represent significant progress toward overcoming spatial resolution limitations in Compton camera imaging.

#### Stochastic origin ensemble reconstruction

Andreyev et al. applied a new reconstruction algorithm, the stochastic origin ensembles (SOE) based on Markov chains for a fast reconstruction of the Compton camera and compared SOE with the list-mode MLEM [120]. They also extended the SOE for resolution recovery from the limited energy and spatial resolutions of detectors [121]. Unlike ML-based reconstruction, SOE does not require the computations of forward and backward projections, and the image is represented by sets of origins of the acquired Compton events instead of voxels. First, new origins of the measured Compton events are randomly generated on conical surfaces where the image from the assigned origins would be an ensemble (*Y*_*S*_) of all possible event origins. Then, the ensemble transition from old to new origins (*Y*_*S*_ → *Y*_*s*+*1*_) for *k*-th event happens by stochastically moving the origin of the event depending on acceptance probabilities, $A\left({Y}_{S}\to {Y}_{S+1}\right)$, which are proportional to the change in event densities, ${D}_{k,s}$.

13 $$ A\left( {Y_{S} \to Y_{S + 1} } \right) \approx min\left( {1, \frac{{D_{k,S + 1} + 1}}{{D_{k,s} }}} \right) $$

**Fig. 8:**
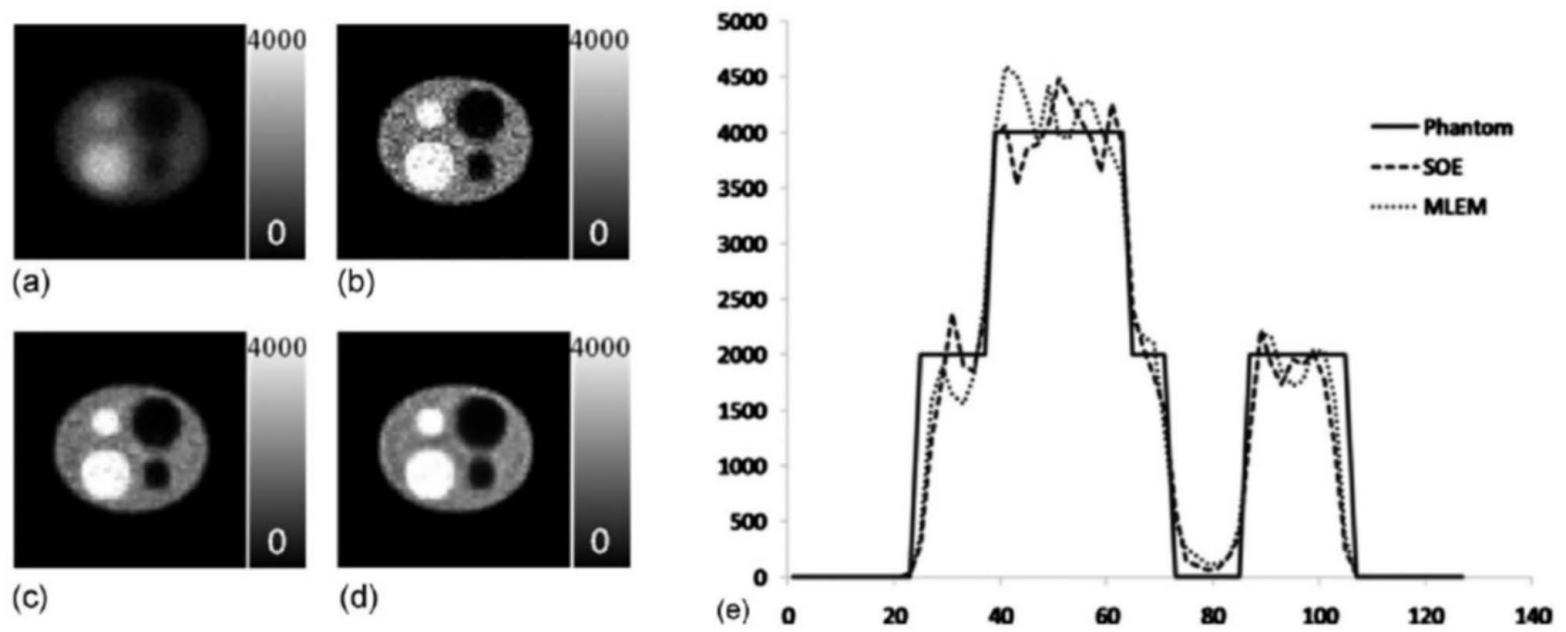
Reconstructed images of a 3D phantom by (**a**) SOE (100 iterations), (**b**) SOE (2000 iterations), (**c**) SOE (2000 iterations) postfiltered with a Gaussian kernel (2.5 mm FWHM), (**d**) MLEM (50 iterations, no postfiltering) and (**e**) the central profiles of SOE and MLEM reconstructions (from [120]; with permission)

During one iteration, the ensemble transition was repeated for all acquired events. After iterations, the ensemble transition will be saturated when the state reaches the quasistationary state. The reconstructed image is voxelized from the ensemble at the final state. Compared to the list-mode MLEM, the postfiltered SOE showed similar image quality while significantly reducing reconstruction time (faster by 4 times in CPU time) (Fig. 8).

#### Deep learning-based approach

Deep learning has revolutionized medical image processing and analysis in many ways [122–128]. Furthermore, recent studies have explored integrating deep learning techniques into Compton camera image reconstruction algorithms to further enhance spatial resolution and image quality. By training neural networks on large datasets of simulated and experimental Compton camera images, the potential of deep learning models to learn complex patterns and correlations in the data has been demonstrated, leading to improved reconstruction accuracy and efficiency. Deep neural networks can directly reconstruct gamma-ray images from raw detector data, potentially improving quantitative accuracy compared to traditional methods. These models can also improve image quality by denoising images, removing artifacts, and enhancing spatial resolution. Furthermore, deep learning approaches can support event classification, helping to accurately identify different types of gamma-ray interactions. Deep learning models can improve robustness and generalization by adapting reconstruction parameters based on input data characteristics.

**Fig. 9:**
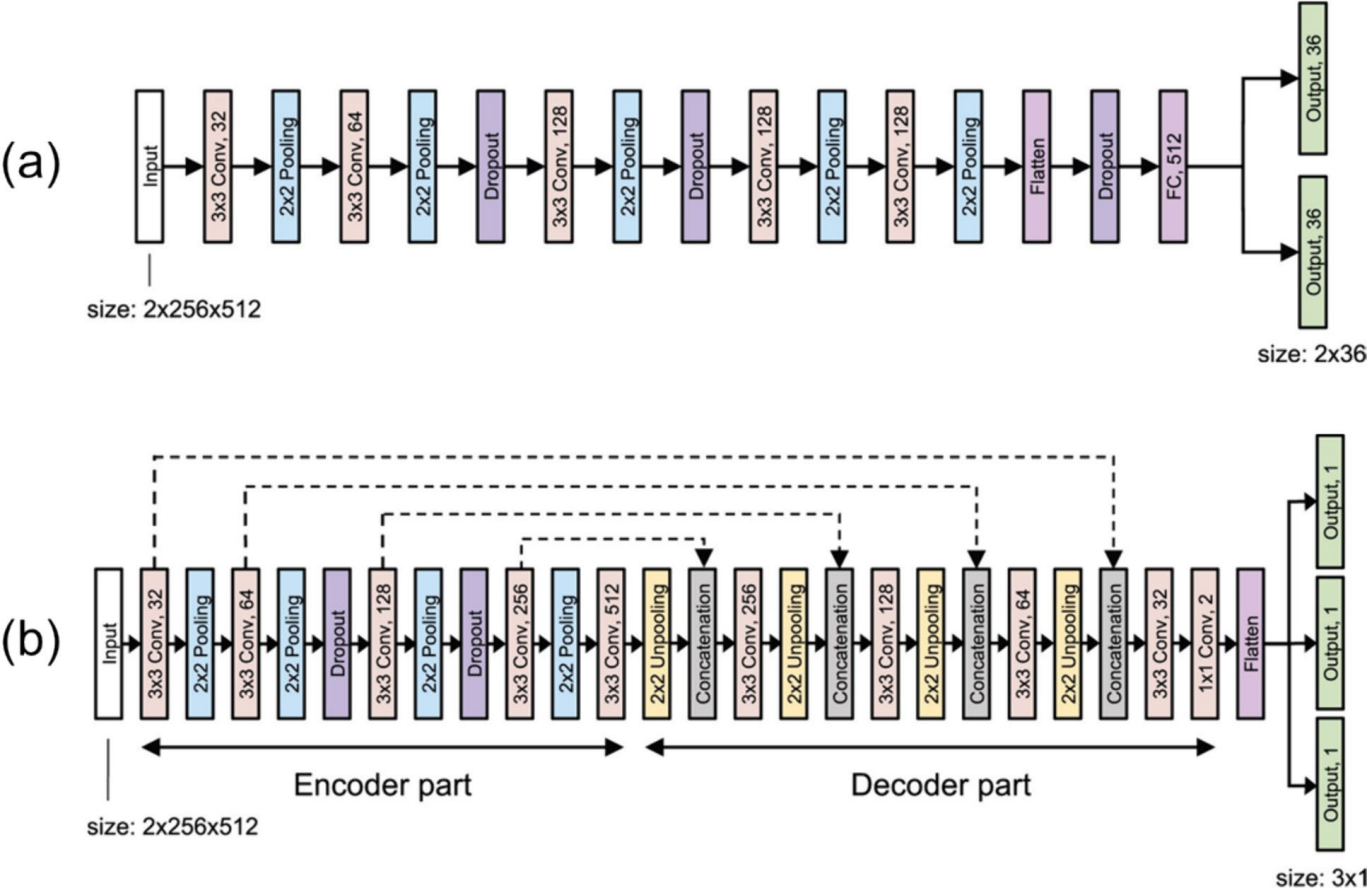
Deep neural network models to predict (**a**) the electron recoil direction and (**b**) the scattering position. (from [129]; with permission)

In the electron-tracking Compton cameras, it is important to accurately determine the scattering position and electron recoil direction. Ikeda et al. employed convolutional neural networks (CNNs) to predict these parameters from track images acquired using a gas time projection chamber (TPC) [129]. This task is challenging because multiple scattering of recoil electrons complicates track images. The CNNs were trained and validated using Monte Carlo simulation data, and their performance was further evaluated using experimental data. Traditionally, in TPC, the scattering position and recoil direction have been determined based on the time difference between the rising and falling edges of signals from the micro-pattern gas detectors (Fig. 9). However, the CNNs outperformed the traditional approaches, improving the PSF of ^137^Cs point source by 32%.

**Fig. 10:**
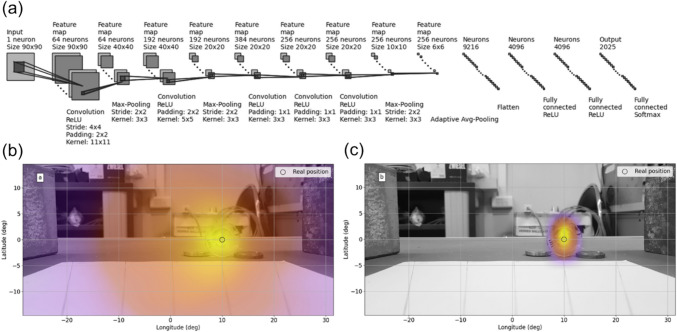
Deep learning-based source position estimation. (**a**) Network architecture, (**b**) Simple back-projection, (**c**) CNN. (from [130]; with permission)

Deep learning also significantly reduced the computation time for image reconstruction and enhanced the sensitivity of a Compton camera based on a single planar CdTe pixelated detector. Daniel et al. trained a CNN to estimate source positions from simply back-projected images of the CdTe detector (Fig. 10) [130]. The network was trained using simulated data and tested with real measurement data using a ^137^Cs source, providing equivalent localization performance to SOE with resolution recovery while substantially reducing computation time.

**Fig. 11:**
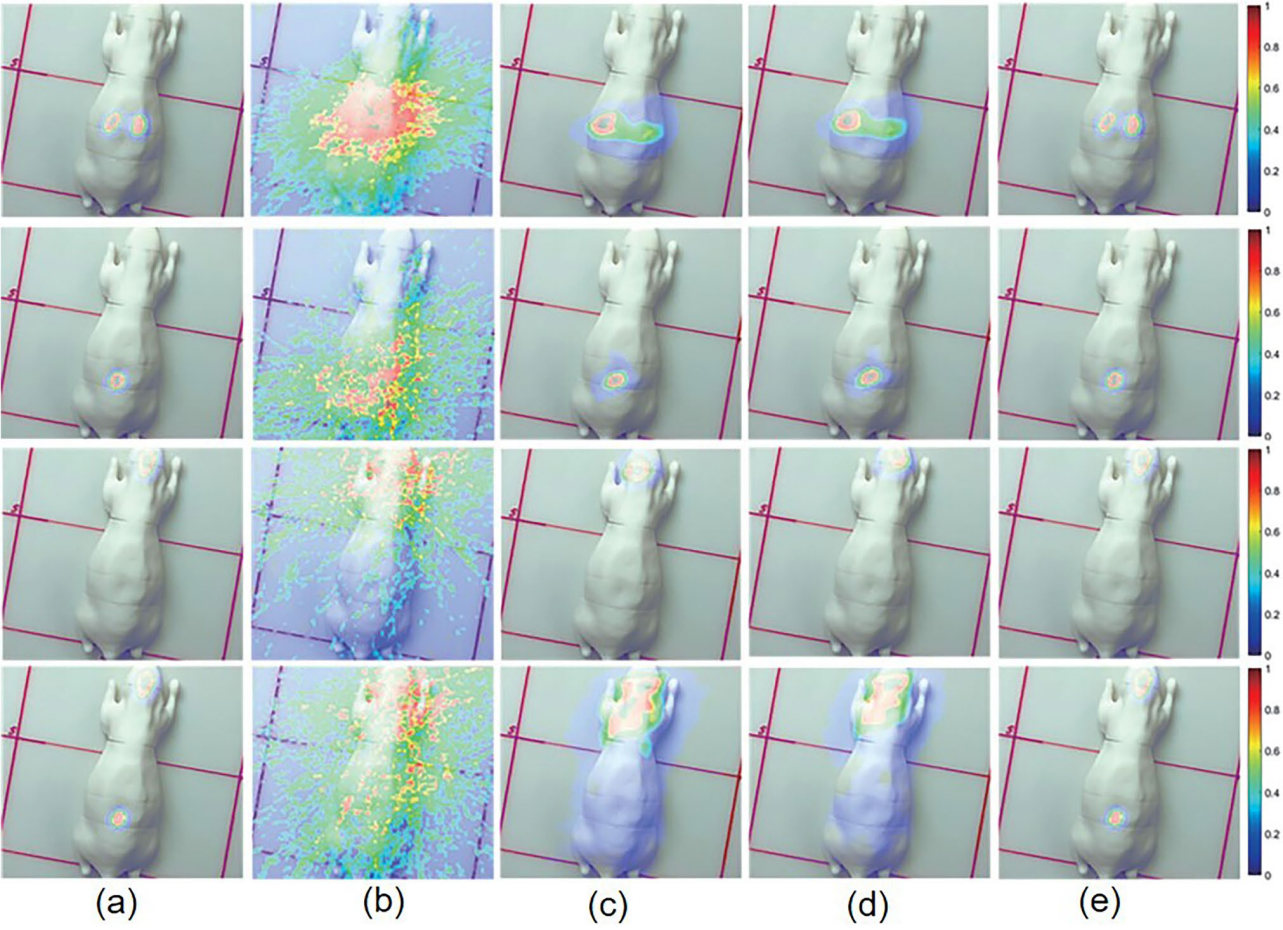
Deep learning-based Compton camera image enhancement. (**a**) ground truth, (**b**) Monte Carlo back-projection, (**c**) list-mode MLEM with resolution recovery, (**d**) origin ensemble with resolution recovery, and (**e**) deep learning-based enhancement of Monte Carlo back-projection. (from [135]; with permission)

Deep learning-based approaches have also proven useful for image enhancement of a 3D-CZT Compton camera. To obtain more accurate reconstruction results from 3D-CZT detector data, Yao et al. applied a U-Net, widely used in medical image processing [131–134], to roughly reconstruct images using Monte Carlo sampling-based back-projection while achieving resolution recovery [135]. In this study, deep learning remarkably improved the image resolution and source localization of a 3D-printing mouse phantom, as shown in Fig. 11.

Considering these advancements, machine learning holds great potential to revolutionize Compton camera technology, particularly in the area of biomedical imaging. One of the key directions is to develop sophisticated reconstruction algorithms tailored for Compton camera image processing, leveraging deep learning architectures like generative models to enhance spatial resolution, image quality, and reconstruction accuracy. Machine learning-based integration with multi-modal data sources, such as computed tomography or magnetic resonance imaging, could enable more comprehensive and accurate biomedical imaging. Real-time processing capabilities, adaptive reconstruction parameters, and task-specific optimization are also areas of focus, and they are expected to improve imaging performance for specific applications such as cancer detection. Furthermore, incorporating uncertainty estimation into machine learning models could provide valuable insights into the reliability of Compton camera imaging results.

## Medical and molecular imaging applications

**Fig. 12:**
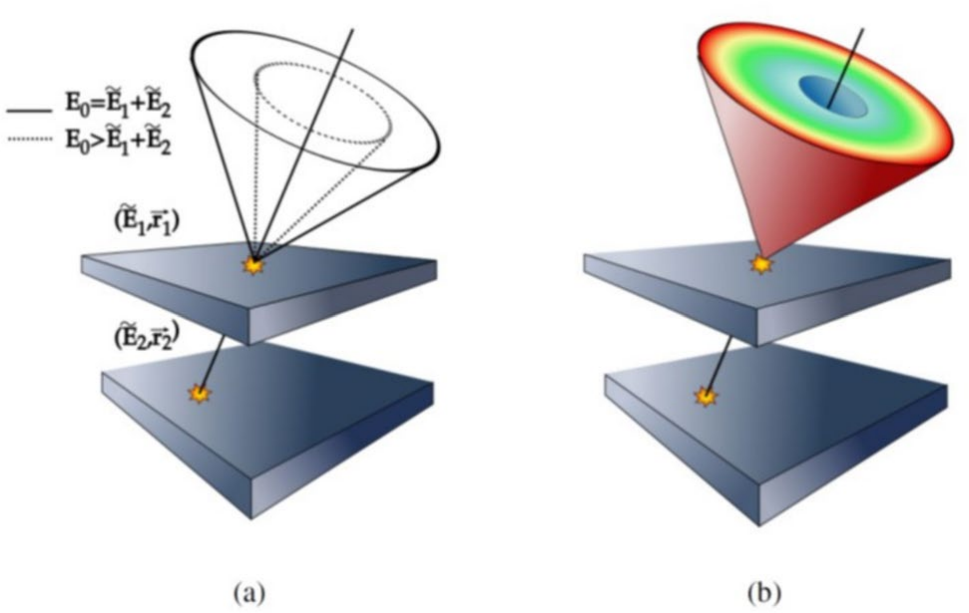
Illustration of (**a**) the possible conical surfaces defined with a range of scattering angles and (**b**) the varying probabilities of different initial gamma energies. (from [141]; with permission)

The Compton camera has been utilized in close-up scans of radioactive source distributions with high sensitivity and spatial resolution. Close-up scans for breast and prostate imaging have been conducted using the Compton camera [136, 137]. In addition, Peterson et al. and Frandes et al. have explored the application of the Compton camera in monitoring prompt gamma rays emitted during proton radiotherapy and hadron therapy, respectively [138, 139]. Krimmer et al. provided a comprehensive review of the utilization of both physically collimated images and electronically collimated Compton cameras for secondary prompt gamma imaging [140]. For prompt gamma imaging, it is important to estimate the initial energies of the measured prompt gammas because it is unknown and has a continuous spectrum. To reconstruct a distribution simultaneously in spatial and energy domains, Muñoz et al. and Roser et al. adopted a list-mode MLEM with a system matrix obtained via Monte Carlo simulations [141, 142]. The system matrix was pre-computed with the simulated physical detection processes of all possible emissions from the measured successive interactions on detectors. Thus, a measured successive detection can define the possible conical surfaces with a range of scattering angles having varying probabilities of different initial energies as shown Fig. 12.

Multi-tracer imaging emerges as a significant biomedical application field of Compton cameras [143]. SPECT can also perform multi-tracer imaging, such as dual-tracer imaging with ^123^I and ^99m^Tc for simultaneous assessment of neurotransmission and cerebral perfusion [144]. However, selecting an appropriate collimator presents challenges for multi-tracer SPECT imaging due to the inherent tradeoff between collimator septal penetration and sensitivity. Collimators optimized for higher-energy gamma rays tend to exhibit decreased sensitivity for lower-energy gamma rays, while those optimized for lower-energy gamma rays often lead to increased septal penetration artifacts for higher-energy gamma rays. This delicate balance complicates the choice of collimator configuration, as it directly impacts the imaging quality and accuracy of multi-tracer SPECT studies. In contrast, Compton cameras, which do not rely on mechanical collimators, are not subject to the septal penetration and sensitivity tradeoff. The high-energy resolution of semiconductor detectors typically used in Compton cameras further enhances their superiority in multi-tracer imaging. These advantages make Compton cameras particularly well-suited for applications requiring simultaneous assessment of multiple radioisotopes with different gamma-ray energies.

**Fig. 13:**
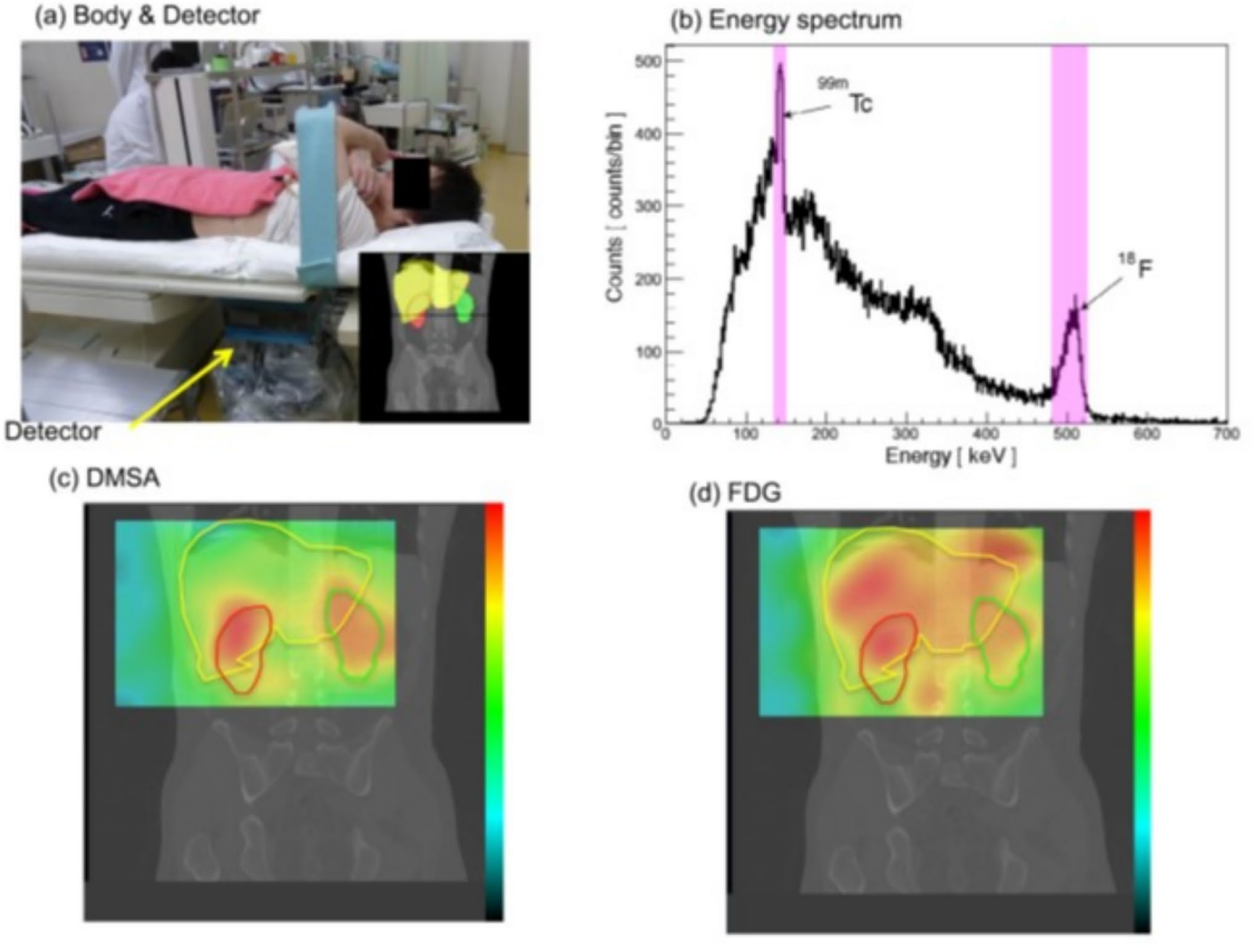
Simultaneous dual-isotope imaging of ^99m^Tc-DMSA and ^18^F-FDG in a human subject using a Si/CdTe Compton camera. (**a**) Experimental setup for dual-isotope imaging, (**b**) energy spectrum showing peaks of ^99m^TC and ^18^F, dual-isotope images of (**c**) ^99m^Tc-DMSA and (**d**) ^18^F-FDG (from [147]; with permission)

The feasibility of the Compton camera for multi-tracer imaging has been demonstrated through several studies utilizing various radioisotopes. Yang et al. conducted the multiple isotope imaging with three different tracers, ^60^Co, ^137^Cs, and ^152^Eu, using a Compton camera equipped with two segmented Ge detectors [145]. Motomura et al. performed simultaneous imaging of (i) ^59^Fe, ^65^Zn, and ^88^Y in a tumor-bearing mouse and (ii) ^65^Zn, ^85^Sr, and ^131^I in a normal ICR mouse [146]. In addition, Fig. 13 shows simultaneous dual isotope images of ^99m^Tc-DMSA and ^18^F-FDG conducted in a human subject using a Si/CdTe Compton camera [147]. These studies underscore the versatility and effectiveness of the Compton camera in multi-tracer imaging across various experimental settings and radioisotopes.

**Fig. 14:**
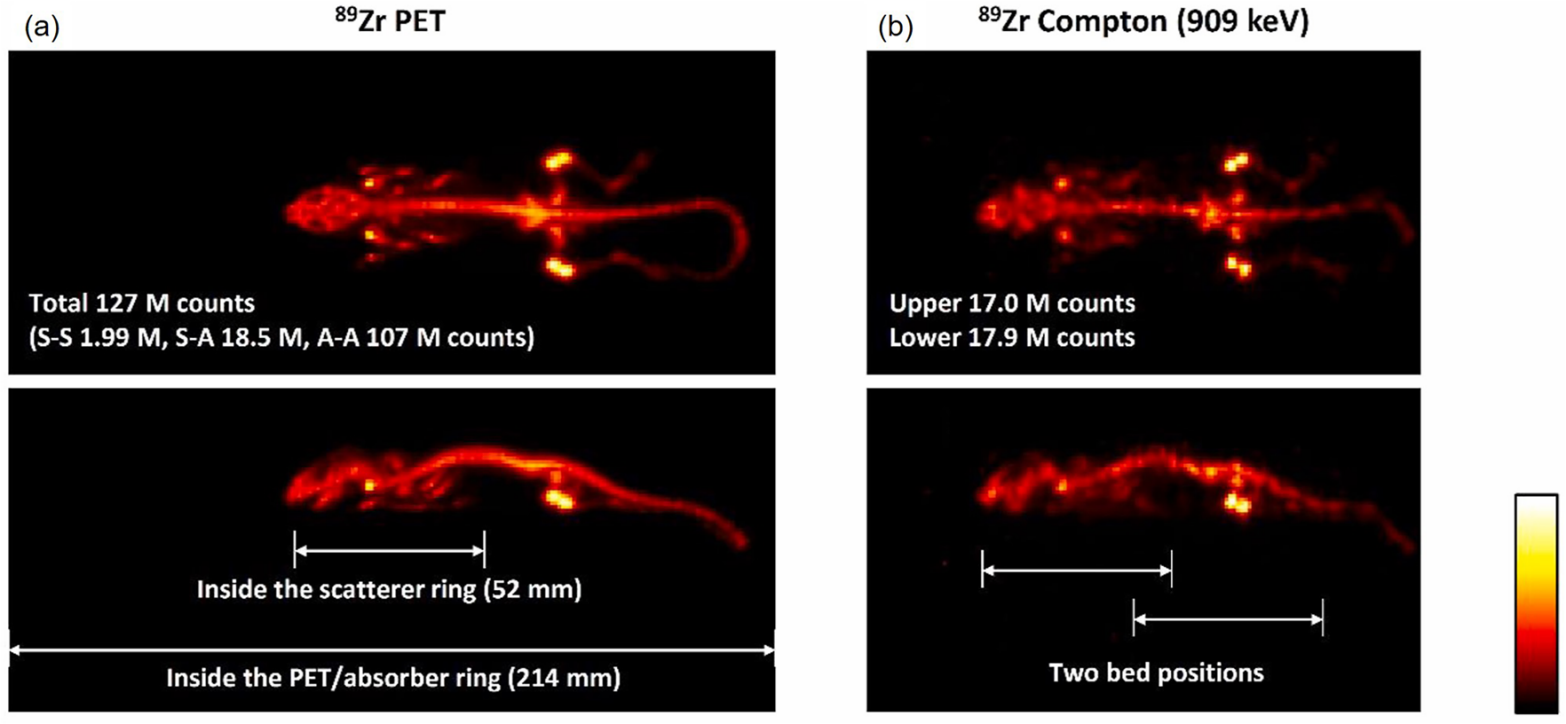
(**a**) PET and (**b**) Compton images of mouse acquired using the WGI system with ^89^Zr-oxalate. (from [148]; with permission)

By integrating the Compton camera with PET technology, the imaging system benefits from complementary detection capabilities, resulting in enhanced overall sensitivity. Yamaya et al. introduced the concept of Whole Gamma Imaging (WGI), a revolutionary system that seamlessly integrates PET and Compton imaging modalities within a single device [148]. This approach enables simultaneous detection and imaging of gamma rays emitted from various sources. In the WGI, composed of two detector rings, the inner ring serves as a scatterer for Compton imaging, while the other ring acts as both an absorber and PET detector. They have demonstrated that the WGI system could produce Compton images of ^89^Zr distributions nearly identical to PET images (Fig. 14). Moreover, it proved valuable for triple-gamma imaging with ^44^Sc.

## Conclusion

In this review, we have provided a comprehensive overview of the various reconstruction methods developed for Compton cameras, highlighting both analytical and statistical approaches. The discussion underscores the significant advancements in image reconstruction techniques that have been tailored to the unique challenges posed by the Compton camera, such as the inherent randomness in photon emission and detection processes.

Analytical methods, while computationally efficient, are often limited by their sensitivity to noise and their inability to fully mitigate the ill-posed nature of the reconstruction problem. Conversely, statistical reconstruction methods, particularly those based on ML and MAP approaches, offer enhanced accuracy and robustness. These methods have shown great promise, especially when combined with advanced techniques like resolution recovery models and high-performance computing, including GPU-based parallel processing.

Moreover, innovations such as the SOE methods and deep learning-based approaches represent significant steps forward, offering faster convergence and improved handling of statistical fluctuations in measured data. The integration of these advanced reconstruction techniques with novel imaging systems further emphasizes the potential of Compton cameras in various applications.

In conclusion, the ongoing development of both analytical and statistical reconstruction methods continues to push the boundaries of what is possible with Compton camera imaging. As computational power increases and new algorithms are developed, the potential for more accurate, efficient, and versatile imaging systems will continue to grow, making significant contributions to fields such as medical imaging, astrophysics, and nuclear security.
